# Single and Serial Fetal Biometry to Detect Preterm and Term Small- and Large-for-Gestational-Age Neonates: A Longitudinal Cohort Study

**DOI:** 10.1371/journal.pone.0164161

**Published:** 2016-11-01

**Authors:** Adi L. Tarca, Edgar Hernandez-Andrade, Hyunyoung Ahn, Maynor Garcia, Zhonghui Xu, Steven J. Korzeniewski, Homam Saker, Tinnakorn Chaiworapongsa, Sonia S. Hassan, Lami Yeo, Roberto Romero

**Affiliations:** 1 Perinatology Research Branch, NICHD/NIH/DHHS, Bethesda, MD, and Detroit, Michigan, United States of America; 2 Department of Obstetrics and Gynecology, Wayne State University School of Medicine, Detroit, Michigan, United States of America; 3 Department of Computer Science, Wayne State University College of Engineering, Detroit, Michigan, United States of America; 4 Department of Epidemiology and Biostatistics, Michigan State University, East Lansing, Michigan, United States of America; 5 Department of Obstetrics and Gynecology, University of Michigan, Ann Arbor, Michigan, United States of America; 6 Center for Molecular Medicine and Genetics, Wayne State University, Detroit, Michigan, United States of America; Johns Hopkins University, UNITED STATES

## Abstract

**Objectives:**

To assess the value of single and serial fetal biometry for the prediction of small- (SGA) and large-for-gestational-age (LGA) neonates delivered preterm or at term.

**Methods:**

A cohort study of 3,971 women with singleton pregnancies was conducted from the first trimester until delivery with 3,440 pregnancies (17,334 scans) meeting the following inclusion criteria: 1) delivery of a live neonate after 33 gestational weeks and 2) two or more ultrasound examinations with fetal biometry parameters obtained at ≤36 weeks. Primary outcomes were SGA (<5^th^ centile) and LGA (>95^th^ centile) at birth based on INTERGROWTH-21^st^ gender-specific standards. Fetus-specific estimated fetal weight (EFW) trajectories were calculated by linear mixed-effects models using data up to a fixed gestational age (GA) cutoff (28, 32, or 36 weeks) for fetuses having two or more measurements before the GA cutoff and not already delivered. A screen test positive for single biometry was based on Z-scores of EFW at the last scan before each GA cut-off so that the false positive rate (FPR) was 10%. Similarly, a screen test positive for the longitudinal analysis was based on the projected (extrapolated) EFW at 40 weeks from all available measurements before each cutoff for each fetus.

**Results:**

Fetal abdominal and head circumference measurements, as well as birth weights in the Detroit population, matched well to the INTERGROWTH-21^st^ standards, yet this was not the case for biparietal diameter (BPD) and femur length (FL) (up to 9% and 10% discrepancy for mean and confidence intervals, respectively), mainly due to differences in the measurement technique. Single biometry based on EFW at the last scan at ≤32 weeks (GA IQR: 27.4–30.9 weeks) had a sensitivity of 50% and 53% (FPR = 10%) to detect preterm and term SGA and LGA neonates, respectively (AUC of 82% both). For the detection of LGA using data up to 32- and 36-week cutoffs, single biometry analysis had higher sensitivity than longitudinal analysis (52% vs 46% and 62% vs 52%, respectively; both p<0.05). Restricting the analysis to subjects with the last observation taken within two weeks from the cutoff, the sensitivity for detection of LGA, but not SGA, increased to 65% and 72% for single biometry at the 32- and 36-week cutoffs, respectively. SGA screening performance was higher for preterm (<37 weeks) than for term cases (73% vs 46% sensitivity; p<0.05) for single biometry at ≤32 weeks.

**Conclusions:**

When growth abnormalities are defined based on birth weight, growth velocity (captured in the longitudinal analysis) does not provide additional information when compared to the last measurement for predicting SGA and LGA neonates, with both approaches detecting one-half of the neonates (FPR = 10%) from data collected at ≤32 weeks. Unlike for SGA, LGA detection can be improved if ultrasound scans are scheduled as close as possible to the gestational-age cutoff when a decision regarding the clinical management of the patient needs to be made. Screening performance for SGA is higher for neonates that will be delivered preterm.

## Introduction

The detection of fetal growth disorders is an important goal in modern prenatal care [1–4]. Sonographic fetal biometry is the method of choice to assess fetal size and growth [5–8]. Yet, there has been considerable controversy about the need for serial biometry measurements during pregnancy [9,10], and at the present time, professional organizations do not recommend this approach [11]. Controversies include: which reference range or standard to use for fetal size and growth (e.g., local vs. international) [12–15], the timing at which fetal biometry should be obtained [16], the need for serial measurements [17], which specific anatomic parameters to monitor (e.g., abdominal circumference, head circumference, estimated fetal weight), and the precise cutoff points to identify abnormalities. Moreover, the diagnostic endpoint is also a subject of controversy. While emphasis has been placed on identification of the small- or large-for-gestational-age infant, some have argued that the ideal end-point should be one at which not only smallness at birth but also morbidity be considered [18–20].

An important issue is whether serial fetal biometry should be used in all pregnancies [21,22]. A prospective cohort study of unselected nulliparous women with a singleton, viable gestation reported that universal sonography in the third trimester almost tripled the detection of SGA neonates compared to clinically indicated sonography (from 20% to 57%) [23]. This improved sensitivity was associated with an increased rate of false positive diagnoses. The current study addresses whether or not serial fetal biometry can improve the detection of SGA and LGA neonates over that of a single fetal biometric examination.

## Methods

This longitudinal study was conducted at the Center for Advanced Obstetrical Care and Research of the Perinatology Research Branch, *Eunice Kennedy Shriver* National Institute of Child Health and Human Development (NICHD), National Institutes of Health (NIH), and Wayne State University, at Hutzel Women’s Hospital, Detroit, Michigan, from January 2009 until December 2014. All patients provided written informed consent and were enrolled in research protocols approved by the Human Investigation Committee of Wayne State University and by the Institutional Review Board of the NICHD.

The study population consisted of 3,971 eligible women with singleton pregnancies without congenital or chromosomal anomalies, who underwent serial ultrasound examination every four weeks until 24 weeks of pregnancy and thereafter every two weeks until delivery. However, since the gestational age at first scan varied among patients and not all patients returned to the clinic on schedule, the number and gestational ages at ultrasound examinations varied among patients Exclusion criteria were: delivery prior to 33 gestational weeks, missing data about gestational age at delivery, fetal gender, and birth weight. To be included in this analysis, subjects must have undergone at least two fetal biometry examinations at ≤36 weeks with biparietal diameter (BPD), head circumference (HC), abdominal circumference (AC), and femur length (FL) measurements. The resulting dataset of 3,440 pregnancies (17,334 scans) was analyzed to predict SGA and LGA outcomes for three different time points: 1) delivery after 28 weeks, 2) delivery after 32 weeks, and 3) delivery after 36 weeks. Subjects were included if they underwent two or more fetal biometric examinations at least three weeks apart prior to delivery.

### Ultrasound examinations

Gestational age was determined by the crown-rump length measurement or by the biparietal diameter during the first ultrasound scan (median gestational age 13.4 weeks; IQR 10–19 weeks). The BPD, HC, AC, and FL measurements were obtained by following the recommendations of the International Society of Ultrasound in Obstetrics and Gynecology [24]. Fetal weight was estimated according to a modified Hadlock formula: log_10_(EFW) = 1.4035+0.0441∙AC+0.177∙FL-0.0037∙AC∙ FL+0.0027∙BPD^2^. [25]

### Outcome definitions

The INTERGROWTH-21^st^ birthweight standards according to gestational age at delivery and fetal sex were used to define SGA and LGA neonates [26]. Since the INTERGROWTH-21^st^ birth weight standards were defined for neonates delivered at 33 weeks or more, we limited the study group herein to those delivering a neonate at ≥ 33 gestational weeks. SGA was defined as a birthweight <5^th^ centile, and LGA was defined as a birthweight >95^th^ centile.

Two approaches described below (single biometry and serial/longitudinal biometry) were used to screen pregnancies under three scenarios that differ by: i) the gestational age cut-off (28, 32, and 36 weeks) up to which ultrasound scans were used for prediction of outcomes, provided that two or more scans were available up to the cut-off and ii) the earliest gestational age at delivery of patients included in the analysis (33^+1^, 33^+1^, and 36^+1^ weeks, respectively). These scenarios allow assessing how SGA and LGA prediction performance changes as more data becomes available prior to delivery. Although the population of patients changes somewhat from one scenario to another the comparison between single biometry and longitudinal analysis is performed on the data from the same patients.

### Screening based on the last fetal biometric examination (single biometry)

**Fig 1:**
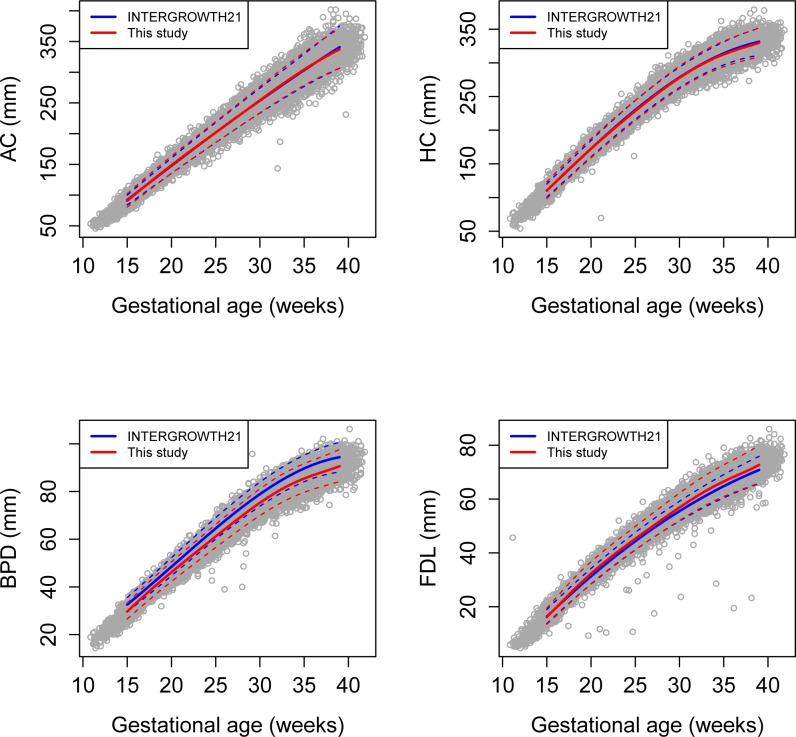
A comparison of this study data and INTERGROWTH 21^st^ standards. Longitudinal measurements of abdominal circumference (AC) (top left), head circumference (HC) (top right), biparietal diameter (BPD) (bottom left), and femur length (FL) (bottom right), as well as 90% confidence intervals, derived from both the Hutzel population used in this study (red) and the INTERGROWTH-21^st^ study (blue).

The first approach used for screening relied on a single (last) EFW evaluation before the GA cut-off under consideration. To account for differences in the GA values at last evaluation between subjects, EFW data was transformed into Z-scores by subtracting the mean and dividing by the standard deviation computed from longitudinal EFW profiles of all fetuses included in this study as previously suggested [27]. The mean and standard deviation of EFW at each GA was derived using a linear mixed-effects model similar to previous work by Royston [28] but using a 5^th^ degree polynomial function instead of fractional polynomials. International standards for EFW were not used for screening due to the differences demonstrated in Fig 1 for BPD and FL between the data collected in this study and INTERGROWTH-21^st^ standards. The INTERGROWTH-21^st^ references were developed based on biparietal diameter measurements from outer-to-outer of the parietal bones, whereas in this study, BPD is measured from outer-to-inner of the parietal bones [29]. The screen status of each patient was set as SGA positive (Z score EFW <z_FPR = 10%_), LGA positive (Z score EFW > z_FPR = 10%_) or appropriate for GA, otherwise. The cut-off z_FPR = 10%_ represents the Z-score percentile that resulted in a false positive rate of 10% for each outcome (SGA or LGA) separately to enable direct comparison between the sensitivity of different prediction methods and cut-off points.

### Screening based on serial fetal biometric examinations (longitudinal)

The procedure to predict an outcome (SGA or LGA) based on two or more longitudinal measurements collected up to a given GA cutoff involved two main steps:

1) For each subject, forecast (extrapolate) the EFW value at term (GA = 40 weeks) from longitudinal determinations of EFW up to the GA cut-off considered using linear mixed-effects models. This step is similar to that reported by Albert [30] implemented in publically available software from the Biostatistics and Bioinformatics Branch of the NICHD, yet adapted for unbalanced data (GA at sample varies among patients), and using a Rossavik [31] instead of a quadratic equation to correlate EFW to GA at examination:

log(EFW)=c+k·log(GA)+s·GA·log(GA)

The linear mixed-effects model included, in addition to the fixed effects coefficients (c, k, s), a corresponding random effect that allowed the trajectory of each subject to depart from the overall population average. The model was fit using the complete dataset to compute fixed effects coefficients, variance components, and properties of the distribution of random effects. For each subject separately, the EFW at two or more time points until the GA cut-off were used to compute subject-specific random effects. Using population-derived fixed effects and subject-specific random effects, the EFW at 40 weeks of gestation was determined (projected).

2) The resulting projections of EFW at 40 weeks GA (EFW_40_) were used to set the screen status of each patient as SGA positive (EFW_40_< c_FPR = 10%_) or LGA positive (EFW_40_> c_FPR = 10%_), where c_FPR = 10%_ is the percentile on EFW_40_ such that the false positive rate is 10% for each outcome (SGA or LGA) separately.

## Results

### Comparison to the INTERGROWTH-21^st^ standards

Demographic characteristics of the population in this study are presented in Table 1. Our patients were mainly African-American women (91.3%) with a high prevalence of BMI>30 (36%) and with a high prevalence of tobacco usage (20%). Of the 3,440 pregnancies included for analysis, 161 (4.7%) were classified as SGA (<5^th^ percentile) and 174 (5.1%) were classified as LGA (>95^th^ percentile) based on the INTERGROWTH-21^st^ birth weight standards.

**Table 1:** Demographic characteristics of the studied cohort.

Characteristic	Study group n = 3440	AGA n = 3105	LGA n = 174	SGA n = 161
Age, years (Mean, SD)	24.1 (5.2)	24.0(5.1)	25.7(5.2) [p<0.001]	24.7(5.8) [p = 0.2]
Height, cm (Mean, SD)	163.0 (7.6) ^1^	163.0(7.5)	165.3(8.1) [p<0.001]	160.7(7.9) [p<0.001]
Weight, Kg (Mean, SD)	76.6 (22.0) ^2^	76.3(21.6)	88.1(26.0) [p<0.001]	70.3(20.4) [p<0.001]
Body Mass Index >30 (n,%)	1242 (36.7%)^2^	1116(36.5%)	82(48.5%) [p = 0.001]	44(28) [p = 0.02]
Body Mass Index >35 (n,%)	666 (19.7%)^2^	584(19.1%)	57(33.7%) [p<0.001]	25(15.9%) [p = 0.26]
Tobacco usage (n,%)	677(19.7)	596(19.2%)	31(17.8%) [p = 0.6]	50(31.1%) [p<0.001]
Nulliparous (n,%)	867 (25.2%)	777(25%)	33(19%) [p = 0.06]	57(35.4%) [p = 0.004]
Preeclampsia (n,%)	190 (5.5%)	157(5.1%)	14(8%) [p = 0.17]	19(11.8%) [p = 0.001]
Chronic Hypertension (n,%)	137(4%)	116(3.7%)	11(6.3%) [p = 0.11]	10(6.2%) [p = 0.14]
Gestational Diabetes Mellitus (n,%)	150 (4.4%)	115(3.7%)	29(16.7%) [p<0.001]	6(3.7%) [p = 0.84]
Preterm delivery (n,%)	357 (10.4%)	315(10.1%)	14(8%) [p = 0.4]	28(17.4%) [p = 0.005]
Birth weight at term, (g) (Mean, SD)	3254g (460g)	3242(372)	4184(287) [p<0.001]	2373(288) [p<0.001]

Data is presented as number (percentage) for binary variable or mean(standard deviation, SD) for continuous variables. P-values for differences in means (two–tailed t-test) or proportions (Fisher’s exact test) are given between SGA vs non-SGA and LAG vs non-LGA.

^1^ data missing for 2 patients.

^2^ data missing for 53 patients.

The rates of SGA were higher among nulliparous, preeclamptic, and women who reported smoking during pregnancy, while being lower in obese women (all p<0.05). The rates of LGA were higher in obese women as well as those with gestational diabetes Mellitus (all p<0.05) (see Table 1).

The 17,334 longitudinal ultrasound measurements for AC, HC, BPD, and FL of the study population are displayed in Fig 1. The number of samples per fetus was 5(3–6), 5(4–6) and 4(3–6) for AGA, SGA and LGA fetuses, respectively [median (IQR)] (Wilcoxon test p = 0.01 for the difference in number of samples per pregnancies between LGA and non-LGA pregnancies). Mean AC and HC derived using linear mixed-effects models for the population in this study matched those published by the INTERGROWTH-21^st^ project [29] with at most 1% discrepancy in the interval of 15 to 39 weeks while the 95% confidence intervals diverged by at most 4%. In the gestational age interval 15–39 weeks GA, the mean of FL was higher by up to 3% while the mean BPD was lower up to 9% in our study population that the one of the INTERGROWTH-21^st^ study, while the 95% confidence intervals diverged by up to 10% for BPD and 7% for FL. These results suggest systematic differences in the protocols for measurements of lengths but similar protocols for circumferences between the current study and INTERGROWTH-21^st^ standards (Fig 1).

### Comparison between single biometry and longitudinal biometric evaluation for detection of small- and large-for-gestational-age fetuses

**Fig 2:**
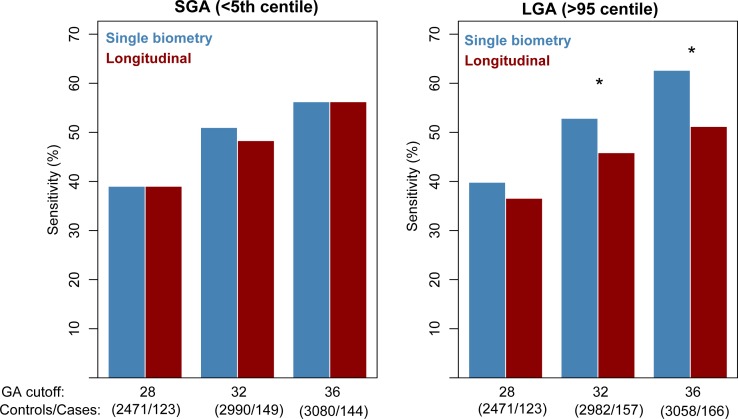
SGA and LGA prediction performance for the entire study population. Sensitivity of predicting small-for-gestational–age (SGA; <5th percentile) (left) and large-for-gestational–age neonates (LGA; >95th percentile) (right) for multiple GA cutoffs. Bars show sensitivity at a 10% false positive rate when using data up to a given GA cutoff (x-axis) to predict the outcome of infants delivered after that cutoff. The number of controls/cases based on which sensitivity is estimated is given under each cutoff. Blue bars correspond to single biometry analysis (last available sample) while red bars are used for longitudinal analysis. * denotes a significant difference in sensitivity for the given cutoff (p<0.05).

Single biometry analysis had a sensitivity of 39%, 51%, and 56% (FPR = 10%) to identify preterm and term SGA neonates delivered after 28, 32, and 36 weeks of gestation based on the last evaluation of EFW available at ≤28, ≤32, ≤36 weeks, respectively (Fig 2). The longitudinal biometry analysis based on projected EFW at 40 weeks from two or more observations before each cutoff resulted in almost identical sensitivities (see Fig 2), (McNemar’s test for correlated proportions; p>0.2 for all cutoffs). Similarly, single biometry analysis had a sensitivity of 40%, 53%, and 63% (FPR = 10%) to identify preterm and term LGA neonates delivered after 28, 32, and 36 weeks of gestation based on the last evaluation of EFW available at ≤28, ≤32, ≤36 weeks, respectively (Fig 2). The longitudinal biometry had lower sensitivity, i.e. 37%, 46%, and 51%, for the same cutoffs, respectively (McNemar’s test; p<0.05 for the 32- and 36-week cutoffs). When data from nulliparous, smoking, obese, preeclamptic, diabetic and chronic hypertensive women was excluded from the analysis, a modest decrease in SGA and LGA prediction performance was noted (AUC decreased up to 0.06, and sensitivity [at 90% specificity] decreased up to 0.15) over the 24 possible combinations of four outcomes (SGA<5^th^, SGA<10^th^, LGA>95^th^, LGA>90^th^) three gestational age cut-off scenarios (28, 32, 36) and two types of analysis methods (single biometry and longitudinal analysis) as shown in S2 Table.

### Impact of gestational age at last sample on prediction performance of SGA and LGA

**Fig 3:**
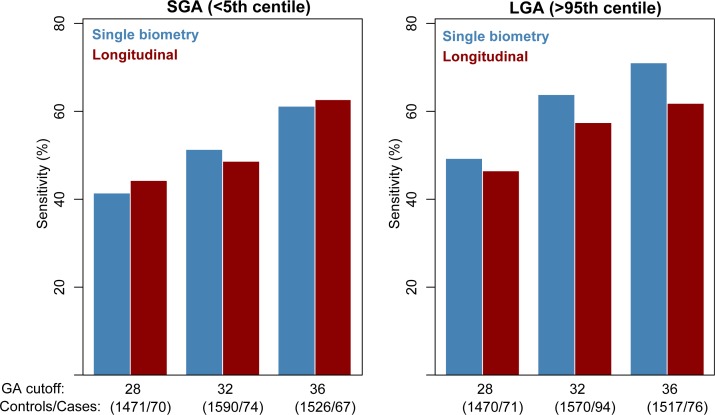
SGA and LGA prediction performance for a restricted study population. Sensitivity of predicting small-for-gestational–age (SGA; <5th percentile) (left) and large-for-gestational–age neonates (LGA; >95th percentile) (right) for multiple GA cutoffs when restricting analysis to subjects with the last measurement before each cutoff within two weeks from the cutoff.

Since ultrasound scans were not scheduled at fixed gestational weeks for each woman, there was substantial variability in the gestational age at the last sample of women included in the SGA and LGA prediction analysis for each of the cutoffs considered (28, 32, and 36 weeks of gestation). Table 2 gives the summary statistics for the last GA value before each cutoff for cases and controls. For example, when the GA cutoff considered was 32 weeks, the median GA at the last point for fetuses that had two or more scans at ≤32 weeks was 30 weeks (IQR: 28–31) for both SGA cases and respective controls as well as for LGA cases and respective controls. To determine the impact of the GA at the last sample on the SGA and LGA prediction performance, we have restricted the analysis to those fetuses having the last scan no more than two weeks prior to the threshold (see Fig 3). The sensitivity of SGA prediction was 41%, 51%, and 61% for the single biometry and 44%, 49%, and 63% for the longitudinal analysis at the ≤28-, ≤32-, ≤36-week GA cutoffs, respectively (FPR = 10%). This corresponds to a net increase in sensitivity of about 5% for both single and longitudinal analyses at the 36-week cutoff only. The sensitivity of LGA prediction in this restricted analysis was 49%, 64%, and 71% for the single biometry and 46%, 57%, and 62% for the longitudinal analysis at the ≤28-, ≤32-, ≤36-week GA cutoffs, respectively (FPR = 10%). This corresponds to a net increase in sensitivity of about 10% for *both* single biometry and longitudinal analysis for *all three* cutoffs in the restricted analysis compared to the overall analysis.

**Table 2:** Distribution of gestational ages at the final evaluation before each gestational age cutoff.

			Controls				Cases		
				GA at last sample				GA at last sample	
Analysis	GA Cutoff	N	Median	25th%	75th%	N	Median	25th%	75th%
SGA	28	2471	26.4	25.0	27.3	123	26.3	25.3	27.1
SGA	32	2990	30.1	28.4	31.1	149	30.0	28.1	31.1
SGA	36	3080	34.0	32.0	35.0	144	33.8	32.3	35.0
LGA	28	2471	26.4	25.0	27.3	123	26.4	24.7	27.3
LGA	32	2982	30.1	28.4	31.1	157	30.3	28.4	31.1
LGA	36	3058	34.0	32.0	35.1	166	33.8	31.4	34.9

N is the number of pregnancies; GA: gestational age

**Fig 4:**
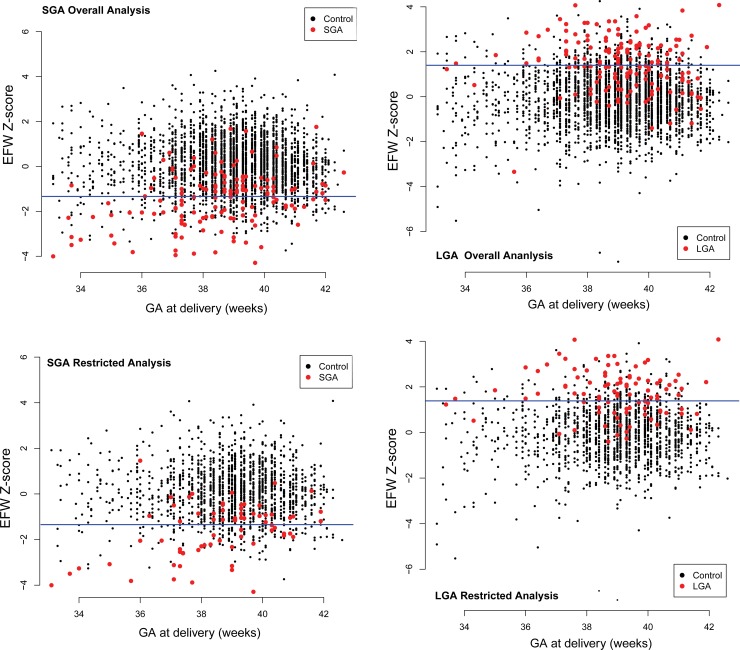
Estimated fetal weight Z-scores as a function of gestational age at delivery and outcome. Estimated fetal weight Z-scores for the last available sample at ≤32 weeks of gestation for all pregnancies (top) and those with the last sample between 30 and 32 weeks (bottom) as a function of gestational age at delivery. Horizontal lines denote the Z-score cutoff that leads to a false positive rate of 10% for each outcome (small- or large-for-gestational-age newborns) separately.

Fig 4 shows the Z-score-transformed EFW value for the last measurement at ≤ 32 weeks as a function of the GA at delivery for both the overall and restricted analyses. The classification model is a cutoff (horizontal line) on the Z-score chosen such that the false positive rate is 10%.

### Effect of gestational age at delivery on the prediction performance of SGA

As shown in Fig 4, the overlap of the EFW Z-scores (last measurement at ≤ 32 weeks) between SGA cases and controls is reduced when delivery occurs earlier. Indeed, when computed separately for preterm neonates (delivery at <37 weeks), the sensitivity for SGA screening was higher (73% at 10% FPR) than for term cases (delivery ≥37 weeks) (46% at 10% FPR) (p = 0.024). The differences in SGA screening performance between preterm and term cases were even more significant and were detected at both the 28- and 32-week cutoffs when the SGA<10^th^ percentile was used as the outcome instead of the SGA<5^th^ percentile. Similar improvements were observed for longitudinal analysis as well (see S1 Table).

### The effect of fetal weight estimation bias

The EFW calculations used in this study for SGA and LGA screening relied on Hadlock’s formula linking birth weight to ultrasound parameters. Since the coefficients in this formula were derived in a different population, biases in fetal weight estimation are known to occur [25] with smaller weights being more underestimated than larger weights (see S1 Fig). As smaller birth weights tend to be obtained at lesser gestational age values, such biases are mitigated by estimating the mean and SD EFW as a function of gestation in the analysis. Therefore, using custom coefficients in Hadlock’s formula had a minor impact on the SGA and LGA prediction performance (see S2 Table).

### Comparison with other studies contrasting single and longitudinal biometry

In order to perform a meaningful comparison between the current study and previous work [30] involving LGA screening delivered after 36 weeks, we have defined LGA as birth weight >4,000g and limited the analysis to those having the last scan after 34 weeks. In this analysis scenario, the single biometry analysis resulted in a sensitivity of 51% (AUC = 0.84), which was slightly higher than the 43% sensitivity (AUC = 0.77) obtained for the longitudinal analysis (p = 0.052). These AUC estimates were in good agreement with those obtained by Albert [30] who reported an AUC of 0.80 for single biometry at 36 weeks and 0.76 for longitudinal analysis based on four observations (including the one at 36 weeks of gestation).

## Discussion

The principal results of this study are as follows:

1. Birthweights in the cohort of 3,440 pregnancies from our study population were similar to those of the INTERGROWTH-21^st^ fetal sex-specific birth weight standards, with 4.7% of neonates being classified as SGA (<5^th^ centile) and 5.1% as LGA (>95^th^ centile).
2. Our locally derived reference for fetal size parameters matched the INTERGROWTH-21^st^ study standards for fetal head and abdominal circumferences (at most 1% discrepancy for mean values and 4% for confidence intervals) but not those for biparietal diameter and femur length (up to 9% for the mean and 10% for confidence intervals).
3. Single biometry (the last available assessment of EFW) as well as longitudinal analysis (from two or more serial measurements of EFW taken three or more weeks apart) at ≤32 weeks can identify one-half of neonates who will be SGA (<5^th^ centile) (FPR = 10%). For LGA prediction at the ≤32- and ≤36-week cutoffs, single biometry is preferable (p<0.05).
4. The sensitivity of single biometry for the detection of LGA increased from 53% to 64% and from 63% to 71% for the 32- and 36-week cutoffs, respectively, when the last scan was within two weeks from the cutoff.
5. Preterm (delivery at <37 weeks) SGA cases are easier to detect based on a single biometry at ≤32 than term SGA cases (73% vs 46% sensitivity at 10% FPR; p<0.05).

### Using universal standards for fetal size evaluation

Universal standards generated by the INTERGROWTH-21^st^ project define optimal fetal size and birth weight based on a population of healthy, well-nourished pregnant women and their fetuses [29]. These standards were proposed for screening for growth abnormalities across all health care systems with the hope that they will improve pregnancy outcomes compared to locally produced references in use worldwide [29]. Although the birth weight distribution of our study population of 3,440 newborns was well-matched to the distribution of birth weight of newborns included in the INTERGROWTH-21^st^ project, this was not the case for two of the four ultrasound parameters (biparietal diameter and femur length). The most likely reasons for discrepancies are differences in the measurement techniques between the two studies (for BPD) and racial differences for femur length [15,32]. Therefore, in this study, we have used INTERGROWTH-21^st^ standards to classify neonates as SGA and LGA but relied on locally derived reference ranges for fetal biometry.

### Single biometry for SGA and LGA screening

Most studies reporting prediction performance of ultrasound-based biometry rely on a single measurement taken at a fixed gestation (e.g., 36 weeks [30,33]) or a gestational-age interval (e.g., 30–34 weeks [16], 34–37 weeks [16], 35–37 weeks [34], 34–42 weeks [27], 30–32 weeks [35], and 34–36 weeks [35]). When the GA at measurement is the same for all subjects, the performance of SGA or LGA prediction can be directly assessed using a cutoff point on the ultrasound parameter value that provides the optimal accuracy or desired fixed specificity [30]. However, when the gestational age at sample varies among fetuses, the ultrasound parameter value or EFW needs to be first converted into a Z-score using an external standard [16,27,34]. This approach was also used in this work to evaluate single biometry.

Performance estimates for single biometry were reported by Souka et al. [16] to be about 50% sensitivity (10% FPR) for both SGA (<5^th^ percentile) and LGA (>95^th^ percentile) for a single EFW evaluation between 30–34 weeks in a Caucasian population of 3,690 women. A similar performance was obtained by Fadigas et al. [34] for SGA prediction in a population of 5,515 patients having an ultrasound scan taken between 35–37 weeks. The authors reported an 89% detection rate for SGA newborns with a 10% false positive rate, combining maternal characteristics, obstetric history, and fetal biometric measurements. Bakalis et al. [36] reported a 65% detection rate of SGA (<5^th^ centile) neonates with delivery at ≥37 weeks when the EFW assessed at 30–34 weeks was combined with maternal factors, uterine artery PI, mean arterial pressure, and serum placental growth factor. The results described herein for single biometry at ≤32 weeks in a mostly African-American population are in very good agreement with these previous findings as we report about a 50% sensitivity for both SGA (<5^th^ centile) and LGA (>95^th^ centile) prediction (FPR = 10%).

A recent study [23] reported screening for fetal growth restriction with universal third-trimester ultrasonography in 3,977 nulliparous women. The authors reported the performance of a single biometry before birth as being 57% (10% FPR) (AUC = 0.87) in detecting SGA <10^th^. For the same outcome (SGA <10^th^) using single biometry at ≤36 weeks, we obtained a 55% sensitivity (AUC = 0.85) (S2 Table). The good agreement between studies is even more relevant considering the differences in the birth weight standards used to define the outcome (INTERGROWTH-21^st^ birth weight standards vs a UK reference), as well as the differences in the distribution of GA at scan for this analysis (median|IQR = 34|32–35, see Table 2, vs 36 weeks).

### Longitudinal biometry for SGA and LGA screening

The primary aim of this study was to evaluate whether the knowledge of fetal-size parameters from prior ultrasound examinations can improve prediction of SGA and LGA neonates compared to the last available fetal biometry examination prior to a given GA cut-off. Previous studies analyzing longitudinal ultrasound data involved either different outcomes and/or asked different questions regarding the benefit of considering longitudinal biometry as a part of clinical care [37,38].

For example, Chang et al [27] reported a study in which weekly ultrasound examinations were performed in 156 women with SGA fetuses based on an ultrasound examination in an attempt to identify those with a reduced ponderal index (weight/length^3^) at birth, and those who will develop suboptimal neonatal outcomes [39]. The information used for discrimination was the rate of change in abdominal circumference and EFW between the first (18–20 weeks) and last (36 weeks) available examinations, after converting these parameters into Z-scores. The authors reported an improved prediction performance using this longitudinal-based approach compared to a single (last available) examination.

Subsequently, Royston [28] described a methodology to construct standards for *growth* vs. size, suggesting that these should be conditional at early measurements in each subject as opposed to one-fits-all growth rate standards. Owen et al. [40] reported weekly growth velocity charts derived from 274 low-risk women examined monthly. The authors [41] studied the effect of scan intervals on predicting infants with anthropometric features of fetal growth restriction; they reported that velocity over 4- and 6-week intervals had a likelihood ratio positive (LR+) of about 10.0 and was better than those derived from a 2-week interval (LR+ about 5.0).

Others have reported that two ultrasound examinations increase the detection of small fetuses and those at a higher risk of perinatal complications; however, the data were treated as separate evaluations, and no growth rate information was used [42].

More recently, a framework to summarize longitudinal biometry by extrapolating individual growth trajectories at a fixed time point in pregnancy (e.g., at term) was proposed [30,43], yet it was limited to term LGA detection and to subjects with four measurements at fixed time points. Our work extends the evaluation of this projection-based approach to longitudinal biometry for preterm and term SGA and LGA screenings under routinely available longitudinal sampling, which involves varying numbers of observations and gestational ages at sampling among subjects. Albert et al. [44] reported that for LGA prediction the last available measurement of AC is more informative than the projected AC value from two or more earlier observations. Our results also suggest that for an outcome like SGA and LGA that are based on birth weight, the most informative variable is the last EFW evaluation, and that especially for LGA detection, the most recent EFW assessment prior to delivery, the higher the detection rate.

### Clinical implications of single or longitudinal biometric fetal evaluations

For preterm and term SGA detection, a single (most recent) or multiple biometry provide similar performance, while for detection of LGA after 32 weeks of gestation, single biometry is preferred [2,45]. For LGA detection, scheduling EFW evaluations closer to the GA cutoff at which a decision needs to be taken increases sensitivity. Since this work was limited to assessing outcomes defined by birthweight, it does not inform on the possible use of serial biometry to detect complications during pregnancy that would help to determine optimal time for steroid administration and the time of delivery. Although several maternal factors presented in Table 1 had a significant association with fetal size and hence risk of an SGA or LGA outcome, the predictive performance of EFW determinations in predicting SGA and LGA was only moderately affected when data from nulliparous, smoking, obese, preeclamptic, diabetic and chronic hypertensive women were excluded from the analysis.

### Strengths and limitations

Among the strengths of this study is the fact that the comparison between single biometry and longitudinal analysis for SGA and LGA screenings was based on outcomes defined using the INTERGROWTH-21^st^ fetus sex-specific birth weight standards as opposed to local reference ranges for birth weight or fixed cutoffs on birth weight. Moreover, by using multiple GA cut-offs, we have assessed the differences between these methods both earlier and later in pregnancy for preterm and term delivered neonates. To these strengths we can add a large sample size of 3,440 women. The main limitation of this study is that the number and gestational age at ultrasound scans varied among patients.

## Supporting Information

S1 Fig Relation between estimated fetal weight and actual birthweight depending whether the original or customized Hadlock formula coefficients are used. Birthweight (g) as a function of Estimated Fetal Weight (g) are shown in pregnancies where the last scan was taken within one week from the delivery. The Hadlock model with original coefficients has a bias, i.e. the smaller the baby the more underestimated is the fetal weight (left). Using a revised formula in which the coefficients are updated for the current population removed the bias (right).
(TIF)

S1 Table Comparison of SGA and LGA prediction performance between term and preterm delivery. GA: gestational age. FPR: False Positive Rate.
(XLS)

S2 Table SGA and LGA prediction performance under additional analysis scenarios. SGA and LGA prediction performance, sensitivity at 10% false positive rate (FPR), and area under the receiver operating characteristic curve (AUC) is shown for the original analysis as well as two other scenarios.
(XLS)
