# Supplementary figures and images for: Single and Serial Fetal Biometry to Detect Preterm and Term Small- and Large-for-Gestational-Age Neonates: A Longitudinal Cohort Study
Source: PLoS One. 2016 Nov 1;11(11):e0164161. doi: 10.1371/journal.pone.0164161 (PMC5089737; doi:10.1371/journal.pone.0164161)

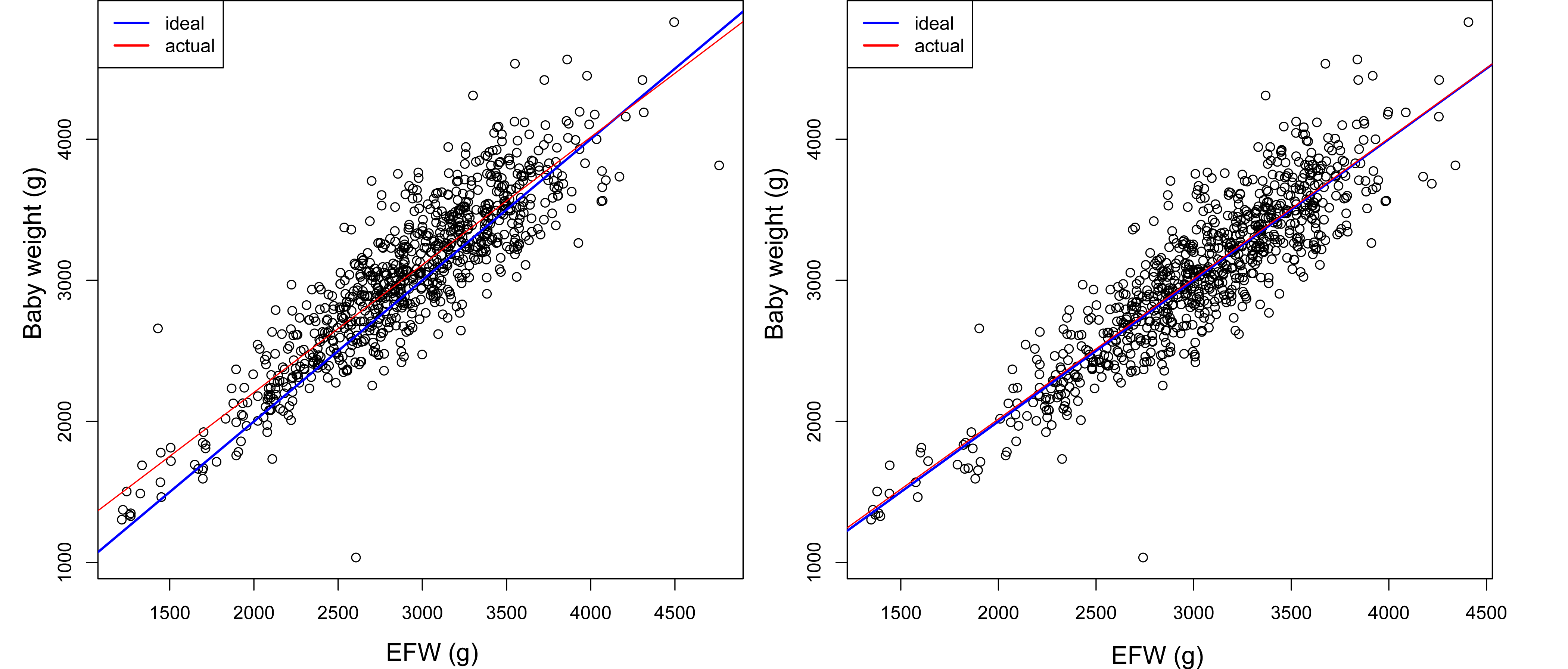

Supplement: S1 Fig — Birthweight (g) as a function of Estimated Fetal Weight (g) are shown in pregnancies where the last scan was taken within one week from the delivery. The Hadlock model with original coefficients has a bias, i.e. the smaller the baby the more underestimated is the fetal weight (left). Using a revised formula in which the coefficients are updated for the current population removed the bias (right). (TIF) [file pone.0164161.s001.tif]
